# Multimodal ECG and biometric data fusion for improved detection of obstructive sleep apnea hypopnea syndrome

**DOI:** 10.3389/fmed.2026.1762868

**Published:** 2026-01-23

**Authors:** Quanjing Zhu, Mingqing Liang, Xingxin Gong, Yong He, Chao Mao

**Affiliations:** 1 School of Automation Science and Engineering, South China University of Technology, Guangzhou, China; 2 Department of Laboratory Medicine, West China Hospital, Sichuan University, Chengdu, China; 3 Clinical Laboratory Medicine Research Center of West China Hospital, Chengdu, China; 4 Sichuan Clinical Research Center for Laboratory Medicine, Chengdu, China; 5 Yanyuan County People’s Hospital, Yanjing, Sichuan Province, China

**Keywords:** obstructive sleep apnea hypopnea syndrome, deep learning, electrocardiogram, LSTM, detection

## Abstract

**Objective:**

Obstructive Sleep Apnea Hypopnea Syndrome (OSAHS) can cause excessive daytime sleepiness and cognitive decline due to long-term nocturnal hypoxia. Without timely treatment, it may increase the risk of obesity, coronary heart disease, stroke, and other serious disorders. However, OSAHS is often underdiagnosed because the standard detection method, overnight polysomnography (PSG), is expensive and available only in limited medical facilities. This study aimed to develop a lower-cost and more accurate approach for detecting OSAHS using electrocardiogram (ECG) signals and biometric data.

**Method:**

We proposed a multimodal feature fusion framework that integrated ECG features extracted through a long short-term memory (LSTM) network with biometric features obtained via support vector machines (SVM). The fused features were classified through a fully connected layer to detect OSAHS. Two independent databases were used to evaluate the performance of the proposed method.

**Results:**

Experimental results showed that the LSTM–SVM fusion model achieved an accuracy of 97.1%, outperforming conventional classification models. In addition, it achieved 92% accuracy on a separate dataset, demonstrating strong generalization ability and potential for practical clinical application.

**Conclusion:**

By combining LSTM-extracted ECG features with SVM-based biometric features, the proposed multimodal fusion method provided highly effective OSAHS detection. The findings suggest considerable potential for the use of this approach in real medical environments.

## 1 Introduction

Obstructive Sleep Apnea Hypopnea Syndrome (OSAHS) is a common symptomatic disorder characterized by dyspnea during sleep. These respiratory interruptions lead to intermittent hypercapnia and hypoxemia, resulting in metabolic dysregulation, endothelial dysfunction, and systemic inflammatory response (1, 2). These biometric changes contribute to various major complications (1–4). OSAHS is a significant global health concern and poses a considerable economic burden. According to estimates, the prevalence of moderate to severe OSAHS in the general adult population ranges from 6% to 17%, while it can reach as high as 49% among the elderly population (3–5). Alarmingly, most of these cases remain undiagnosed (6). Prompt detection and treatment of OSAHS are crucial to prevent the development of various health complications. Unfortunately, a significant number of patients with clear symptoms of OSAHS are unaware of their condition due to a lack of recognition (7).

The gold standard of determining the severity of OSAHS requires overnight laboratory polysomnography (PSG) (8), which gauges the patient’s condition by tracking various symptoms during sleep. PSG signals include Electroencephalogram (EEG), eye movement, ECG, electromyogram (EMG), peripheral oxygen saturation (SpO2), mouth and nose airflow signal, and pharyngeal vibration signal (9, 10). The detection principle of PSG is counting the number of apneic and hypopneas events per hour of sleep, known as the apnea-hypopnea index (AHI). OSAHS can be divided into four categories according to the value of AHI: normal (AHI ≤ 5), mild (5 < AHI ≤ 15), moderate (15 < AHI ≤ 30) and severe (AHI > 30) (11). When using PSG for OSAHS detect, a large number of cables and sensors need to be connected to the subject’s body, and the testing is expensive and inconvenient, making it unable to be widely publicized (12). Furthermore, according to numerous research (13, 14), PSG-based assessment of sleep apnea severity is flawed because it requires wearing the devices for a night in the hospital, where an unfamiliar environment can affect sleep conditions and directly affect measurement results. Moreover, the number of medical institutions and specialists capable of detecting sleep apnea is relatively small (15, 16), which is extremely detrimental to the detection and treatment of OSAHS. As such, it is critical to investigate more convenient and cost-effective detection approaches for OSAHS.

In previous research, people have tried to find various biometric features to identify OSAHS that are easy to detect, such as EEG, ECG, and SpO_2_, etc., (17, 18). Among these, ECG has been extensively utilized as a fundamental tool and a significant indicator in OSAHS detection ECG (19, 20), a technique that captures the electrical activity pattern of the heart during each cardiac cycle, has emerged as a valuable tool in cardiovascular disease detection. ECG signals are represented as a random variable through the arrangement of statistical indices in their sequential occurrence. Mashrur et al. (21) proposed a scale graph-based convolutional neural network (SCNN) to detect OSA using single-lead ECG signals. This study segments nocturnal ECG signals into apneic and normal phases and applies a classification model to distinguish between them, yet this approach does not directly identify OSAHS. Similarly, Sharma and Sharma (22) used four different types of classifiers to classify apneic and normal phases. Both methods use the same dataset as our study, but they focus on classifying ECG signal segments to identify apnea and normal periods. Although the classification results can help identify the state of a specific segment, they cannot directly diagnose or identify whether the patient has OSAHS. Sharma et al. (23) developed an innovative and portable OSA-CAD system utilizing a single-channel ECG to identify apneic and normal ECG events of the duration of per-minute collected from recordings. This method uses classifiers such as K nearest neighbor (KNN), decision tree, linear regression, logistic regression and SVM (24) to classify the extracted ECG features. Among them, SVM is significantly better than others. Indeed, the calculation of the AHI requires a complete overnight ECG or PSG recording, and conventional automated approaches still rely on segment-level apnea event detection followed by event counting. Such pipelines not only increase computational complexity but also suffer from cumulative error propagation across stages. The above-mentioned studies mainly classify short ECG segments (e.g., minute-level or window-level) into apneic and non-apneic states. Although this strategy is useful for apnea event analysis, it does not directly provide a subject-level detection decision, and additional AHI estimation or manual interpretation is still required to determine whether a patient has OSAHS. In contrast, our study adopts a record-level classification strategy: instead of detecting apnea episodes or computing AHI explicitly, we use the complete overnight ECG signal together with biometric information as multimodal input to directly classify whether a subject belongs to the OSAHS category. This formulation simplifies the screening and evaluation workflow, rather than replacing the detection pipeline and offers a complementary, clinically practical alternative to AHI-based diagnosis, while still being consistent with the clinical definition of subject-level OSAHS screening. Therefore, we hope to detect OSAHS by classifying only ECG signals using deep learning methods such as convolutional neural network (CNN), recurrent neural network (RNN), and LSTM, and it can be used as an ablation experiment to compare the experimental results with the method proposed in this article, highlighting the advancement of our method.

Moreover, numerous body measurements have been found to be associated with increased susceptibility to OSAHS. These measurements include body weight, body mass index (BMI), central body fat distribution, and larger neck and waist circumferences, all of which may be considered potential risk factors (1, 5). In this paper we refer to these measurements as biometric data. Furthermore, advanced age and male sex have also been identified as additional risk factors for OSAHS. Despite epidemiological evidence linking age, sex, and BMI with the severity of OSAHS, studies focusing on these three variables for assessing OSAHS risk are scarce and lack depth. Kuan et al. (25) proposed a logistic regression model that utilizes biometric data: age, sex, and BMI, for detecting OSAHS. However, the model’s accuracy on the validation set was found to be only 76.3%.

In summary, although both biometric data and ECG signals have been shown to be effective in OSAHS-related analysis, existing evidence indicates that neither modality is sufficient when used in isolation. Recent deep learning studies have further demonstrated that ECG-based representations can capture apnea-related temporal dynamics with high discriminative potential, particularly when long-sequence temporal models or attention-based architectures are employed. For example, several works in the past three to five years have explored deep neural networks for sleep-apnea–related pattern learning from ECG and cardiorespiratory signals, reporting improved recognition performance and enhanced robustness to signal variability (26, 27). These findings suggest that data-driven temporal representation learning has become an increasingly important direction in OSAHS detection research, although most of these studies still rely on single-modality physiological input and do not incorporate subject-level biometric risk information (28, 29).

However, in this study, combining two types of data (ECG and biometric data) for detecting OSAHS can not only improve the detection accuracy but also improve the generalization of the model. There have been many multimodal studies on ECG signals. Meanwhile, multimodal biometric fusion has also been explored in other application domains. For instance, Ahmad et al. (30) used different methods to convert raw ECG data into three different images to be used as input to CNN. Features are then extracted from the penultimate layer of the neural network and fused. These informative features are ultimately used in a support vector machine (SVM) classifier for ECG heartbeat classification. Regouid et al. (31) proposed a multimodal biometric system that integrates ECG, ear, and iris features for identity recognition based on local descriptors and feature-level fusion. Although this work demonstrates the effectiveness of multimodal fusion for improving biometric identification reliability, its primary objective is person authentication rather than disease screening or medical diagnosis, and the selected modalities mainly reflect morphological and structural identity traits rather than physiological or pathological characteristics. In contrast, the present study focuses on OSAHS detection at the subject level and intentionally combines two conceptually different but clinically meaningful sources of information: dynamic temporal patterns extracted from overnight ECG signals and stable anatomical and metabolic risk indicators represented by biometric data such as age, BMI, and body weight. The proposed fusion strategy is therefore designed not for identity discrimination, but for modeling the complementary relationship between physiological responses and risk-factor profiles in the context of OSAHS screening. ECG can also be combined with data such as fingerprints and finger veins (32, 33) for multi-modal fusion. Therefore, combining and integrating multiple sources of data, such as ECG signals and biometric data, enhances the accuracy and reliability of OSAHS detection methods. This multi-modal approach allows for a more comprehensive analysis of the condition, leading to improved effectiveness in OSAHS detection systems.

Building upon these considerations, we propose a multimodal feature fusion framework (LSTM–SVM) for OSAHS detection, in which ECG sequence features learned by LSTM are combined with biometric features extracted via SVM and adaptively fused for subject-level classification. This formulation differs from prior multimodal biometric recognition systems in both task objective and fusion rationale, as our method is specifically oriented toward medical decision support and aims to enhance detection generalization by integrating heterogeneous, clinically interpretable feature sources. The final experimental results further confirm that the proposed approach achieves superior performance compared with conventional single-modal and non-adaptive fusion models.

Our contributions can be highlighted as follows:

1. To the best of our knowledge, this is the first study to use multi-modal feature fusion method to detect OSAHS.
2. Normalized techniques are used to process ECG signals and biometric feature signals, and corresponding features are extracted using LSTM and SVM, respectively. The experimental results show that our methods retain most of the characteristics of the original data and are beneficial to detecting OSAHS.
3. A feature fusion method involving adaptive weighting for weighted summation is proposed. The fused features are ultimately categorized through a fully connected network layer to obtain classification results.

The rest of the paper is organized as follows. Section 2 describes materials and methods in detail. Section 3 presents the experiments. Section 4 presents the results and discussion. Finally, Section 5 concludes the paper.

## 2 Materials and methods

### 2.1 LSTM

**Figure 1:**
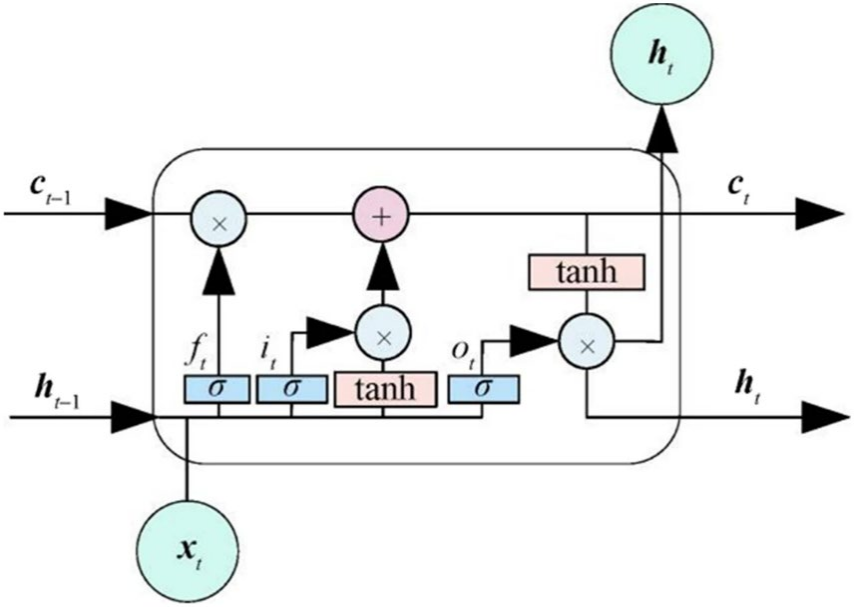
LSTM unit structure.

The original LSTM paper appeared in 1997 (34). This is a temporal recurrent neural network, and LSTM was specifically created to solve the long-term dependency problem of RNN (35–37). As the length of the sequence increases, it encounters the issues of vanishing gradient and exploding gradient. However, LSTM, a variant of RNN, solves these problems by combining the following components: Forget gate (f_t_), which decides what information to discard from the cell state, calculated by Equation 1. Input gate (i_t_), the gate determines which new information will be stored in the cell state, calculated by Equation 2. The update of the storage state unit (c_t_) combines the information in the first two steps, discards the old state, and adds new state information, calculated by Equation 3. The output gate (o_t_) determines what will be output based on the cell state, and h_t_ is the hidden state at the current time step, calculated by Equation 4 and Equation 5. As shown in Figure 1, these gates enable the LSTM to regulate the retention, forgetting, and updating of information. The calculations are performed as follows:

$$f_{t}=\sigma (W_{f}\cdot [h_{t-1},x_{t}]+b_{f})$$ (1)

$$i_{t}=\sigma (W_{i}\cdot [h_{t-1},x_{t}]+b_{i})$$ (2)

$$c_{t}=f_{t}⊙c_{t-1}+i_{t}⊙\tanh(W_{c}\cdot [h_{t-1},x_{t}]+b_{c})$$ (3)

$$o_{t}=\sigma (W_{o}\cdot [h_{t-1},x_{t}]+b_{o})$$ (4)

$$h_{t}=o_{t}⊙\tanh(c_{t})$$ (5)

In the equations above, σ represents the nonlinear activation function, *W* denotes the weight matrix, *b* represents the bias term, x_t_ represents the input vector at time t, h_t-1_ corresponds to the output at the previous time step, and c_t-1_ represents the hidden state at the previous time step.

### 2.2 SVM

Support Vector Machine (SVM) is a supervised learning algorithm instrumental for classification tasks and applicable to regression problems (38). Fundamentally, SVM aims to determine the optimal separating hyperplane which maximizes the margin between different classes in the feature space. One of SVM’s key features is its use of kernel functions to tackle non-linear relationships, allowing the algorithm to conduct operations in a high-dimensional space without explicit transformation of the inputs. This capability renders it powerful for various complex datasets. SVM have shown remarkable performance in diverse domains (39, 40), including image recognition, bioinformatics, and text categorization, underpinning their extensive adoption in practical machine learning challenges.

### 2.3 Dataset

To validate our work, we tested it in the Apnea-ECG Database (41). The data consist of 70 records, divided into a training set of 35 records and a test set of 35 records. And according to the AHI value provided by the database, records with AHI greater than 5 were classified into the OSAHS category, and records with AHI less than or equal to 5 were classified into the normal category. The duration of each recording ranged from little under 7 hours to about 10 hours. Every recording comprises of an ongoing digitalized ECG signal, a collection of machines generated QRS annotations, and a set of apnea annotations (made by human specialists based on simultaneously recorded respiration signals and associated data). There is also a file in the database of about 70 records of biometric data, including height (cm), weight (kg), age, BMI (kg/m^2^), and sex. In this paper, we used the ECG signals and biometric data in the database.

To further validate the generalization capability of the model, we selected the University College Dublin Sleep Apnea Database (42) for additional testing. This database includes 25 participants who were randomly chosen from patients referred to the sleep disorder clinic at St. Vincent’s University Hospital in Dublin. All subjects were at least 18 years old, had no known cardiac conditions or autonomic dysfunctions, and were not taking any medications that interfere with heart rate. The demographic information of the two databases is shown in Table 1.

**Table 1:** Demographic information table.

Variable	Apnea-ECG database	UCD Sleep Apnea Database
Gender	Male: 57	Male: 21
	Female: 13	Female: 4
Age	45.1 ± 10.8	50 ± 10
Height	175.8 ± 5.6	173.3 ± 9.6
Weight	86.8 ± 20.7	95.0 ± 14.7
BMI	28.0 ± 6.5	31.6 ± 4.0

### 2.4 LSTM-SVM

Feature extraction is a vital process in machine learning and deep learning. In this process, dimensionality reduction technology is often used to reduce the number of features and help reveal patterns and structures that may be hidden in the data, thus playing an important role in feature extraction. Dimensionality reduction transforms high-dimensional datasets into more compact, lower-dimensional representations, thereby alleviating computational load and fostering model generalizability. This technique is essential for distilling critical features and patterns from raw data, while dispensing with extraneous or repetitive information. Good feature extraction significantly enhances model performance, enabling better classification, regression, and clustering (43–45).

Through the previous analysis, it is evident that ECG signals and biometric data encode complementary physiological information relevant to OSAHS. The ECG signal reflects autonomic regulation, cardiorespiratory coupling, and sleep–related heart rate modulation across time, whereas biometric data (e.g., age, BMI, weight, and neck circumference) capture stable anatomical and metabolic risk factors that are strongly associated with OSAHS severity. Due to this intrinsic heterogeneity, the two modalities contribute unequally to diagnosis under different patient conditions (e.g., obese vs. non-obese subjects, mild vs. severe OSAHS). Therefore, rather than simply concatenating the two feature vectors, we designed a feature fusion classification framework with adaptive weighting, which allows the network to automatically learn the relative contribution of ECG-derived dynamic features and biometric structural features at the subject level.

**Figure 2:**
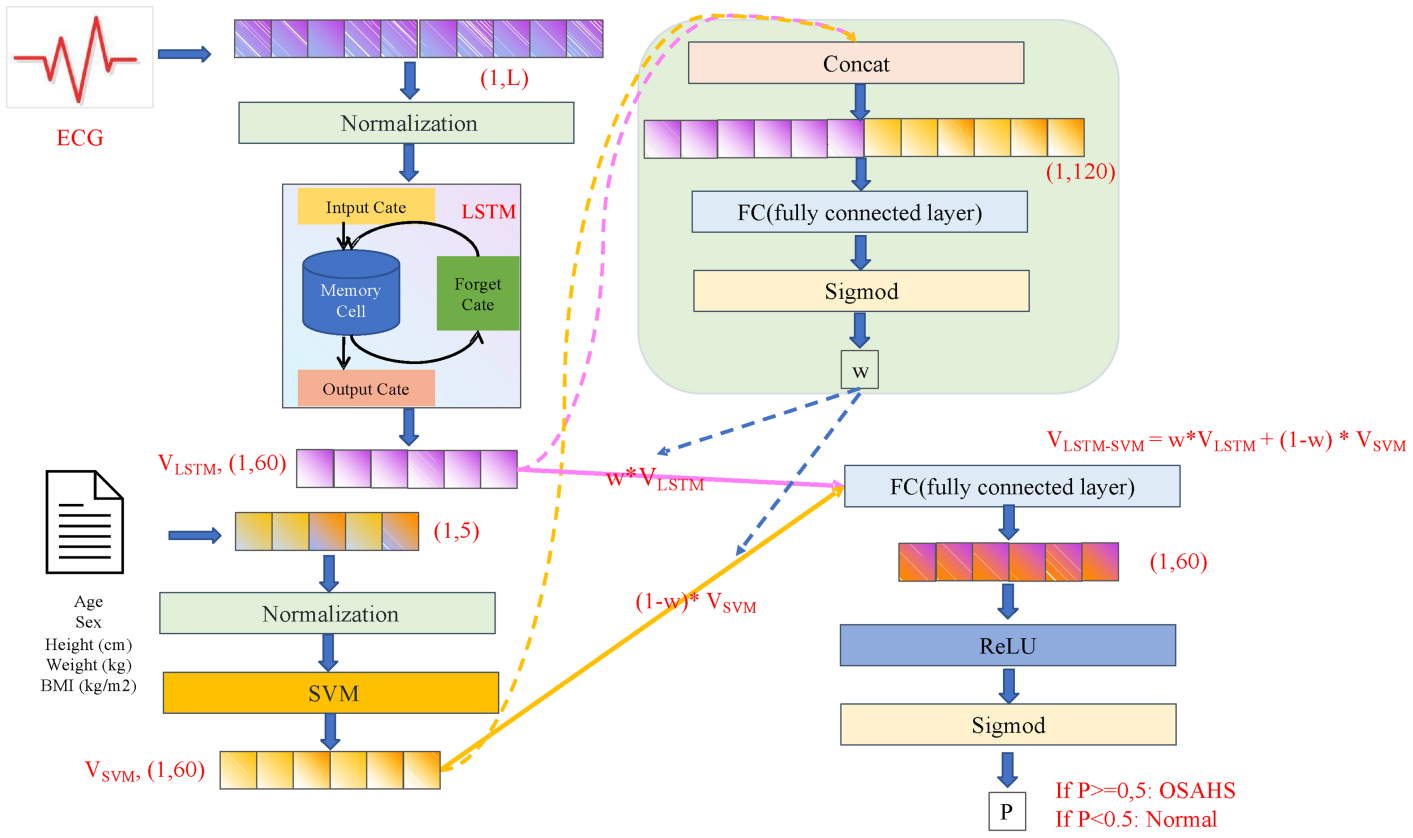
The overall architecture of LSTM-SVM. This structure can be divided into four parts: (1) the upper left part is the extract features of ECG signals using LSTM model, (2) the lower left part is the extract features of biometric data using SVM model, (3) the upper right part is the automatic update of the weights of the fusion features, and (4) the lower right part is the automatically update the weights of fused features.

As shown in Figure 2, the preprocessed ECG signal is fed into the LSTM network, which captures long-term temporal dependencies and sequence-level variations across the overnight recording, yielding a feature vector $V_{\text{LSTM}}$ of dimension (1, 60). The biometric data are processed via SVM to produce a compact feature vector $V_{\mathrm{SVM}}$ of dimension (1, 60), representing static physiological risk attributes. To integrate the two modalities, we introduce an adaptive weighting module. The concatenated feature vector is passed through a fully connected layer and a sigmoid activation to obtain a learnable weight w∈[0,1] , which controls the relative contribution of each modality during fusion. Then the new feature vector V_LSTM-SVM_ is obtained through Equation (6), and the weighted fusion of adaptive weights is completed. Unlike simple concatenation or fixed-ratio fusion, this mechanism enables the model to assign higher weights to ECG features when temporal abnormalities dominate (e.g., strong apnea-related heart rate fluctuations), or to prioritize biometric factors when anatomical risk markers are more informative. The weight w is optimized via back-propagation during training, allowing the fusion process to adapt to inter-subject variability and reduce modality bias.

$$V_{\text{LSTM}-\mathrm{SVM}}=w^{*}\;V_{\text{LSTM}}+{\left(1-w\right)}^{*}\;V_{\mathrm{SVM}}$$ (6)

This design not only enhances feature complementarity but also mitigates over-reliance on a single modality, which is particularly important in heterogeneous clinical populations.

Through the feature extraction process, the obtained feature vector V_LSTM- SVM_ passes through the ReLU activation function and then uses the sigmoid function to map the value, limiting it to the range of 0 to 1. Such an operation converts the feature vector V_LSTM-SVM_ into a numerical value P representing probability. When the value of P is greater than or equal 0.5, it can be concluded that the piece of data is in the OSAHS category. On the contrary, when the value of P is less than 0.5, it can be inferred that the piece of data does not belong to the OSAHS category.

## 3 Experiment

To comprehensively evaluate the effectiveness of the LSTM-SVM model proposed in this study, we designed several sets of comparative experiments. First, in the model structure, to explore the contribution of different components in the model, we replaced LSTM with CNN and RNN to construct CNN-SVM and RNN-SVM models to compare the impact of various deep learning models on feature extraction. Secondly, we conducted ablation experiments to clarify the specific contribution of feature fusion to the overall performance. In the ablation experiments, we used LSTM, CNN, or RNN to extract ECG signal features separately without combining biometric data for classification. These experiments help evaluate the model’s performance without feature fusion, to analyze the actual contribution of fused features. Through these analyses, we can more accurately summarize the importance of feature fusion and the impact of each model component on model performance, further proving the effectiveness and superiority of our model in handling complex biological signal classification tasks. For ECG preprocessing, the entire overnight sequence of each subject was processed as a single sample for feature learning. No temporal slicing or segment-level sampling across subsets was performed. In particular, ECG segments from the same subject were never split across training and testing sets, ensuring that all reported results reflect cross-subject generalization.

### 3.1 Data processing

In this database, the ECG acquisition equipment obtains a complete ECG signal by recording the signal 100 times per second. For each recording period of seven to ten hours, we obtained a very long sequence of data. Then, we uniformly crop the sequence according to the length of eight hours and fill in 0 through padding if it is less than 8 h. We then normalized the data, adjust the scale and range of the data, and eliminate scale differences. This makes it easier to extract complete features through LSTM. For biometric data, SVM was used to extract features after the same normalization process.

### 3.2 Model training

In the experiment, we used the Adam optimizer to train the model. Adam (46) uses an adaptive learning rate method and combines the concept of momentum. Adam excels in the gradient descent algorithm by constantly adjusting the learning rate based on the first and second moment estimates of the parameters. To evaluate the performance of the model, we chose the binary cross-entropy loss function (BCELoss) (47). BCELoss is a common loss function used for binary classification problems. It can measure the difference between model predictions and true categories and can be a good measure of the fit of the model. We set the learning rate to 0.001, which is a commonly used initial learning rate. After 50 rounds of training, we observed that the model has reached a convergence state, that is, the loss function of the model has stabilized at a relatively low value, and further training will not bring significant performance improvements.

Our models are all built under Python 3.7 and PyTorch 1.13. When training the model, we use CUDA 12.0 to use the computing power of the GPU to accelerate the training process. The entire training process was completed on Ubuntu 20.04 equipped with 32GB RAM, a 10-core Intel Core i5 processor, and an NVIDIA 4070TI graphics card. These hardware configurations can effectively support the training of deep learning models.

### 3.3 Evaluation metrics

We utilize essential evaluation metrics such as accuracy, precision, recall, F-score, and the confusion matrix to assess the efficacy of the predictive analysis. Accuracy is characterized as the ratio of true predictions to the overall number of predictions made. The precision rate is specifically the measure of correctly predicted positive observations relative to the total predicted positive observations. The recall rate refers to the fraction of actual positives that the model accurately identifies, representing the model’s capacity for detecting the relevant category. The F-score is introduced as a harmonizing metric that incorporates both precision and recall, offering a singular measure reflective of the model’s balance between the two. The Equations 7–9 are the calculation methods of different indicators.

P=TP/(TP+FP) (7)

R=TP/(TP+FN) (8)

F=2∗PR/(P+R) (9)

P is the precision rate, and R is the recall rate. TP denotes the right prediction in terms of the number of correct classes; FP is the incorrect prediction in terms of the number of correct classes. F-score is the harmony between the precision rate and the recall rate average values, and FN is the right prediction in terms of the number of incorrect classes.

The output of a classifier is summarized using a confusion matrix. A k x k table is used to keep track of the classifier’s classification outcomes for k-ary classification. The total amount of data in each row indicates how many instances of this category there are in the data, and each row represents the true attribution category of the data. The total number of each column in the confusion matrix denotes the number of data points that are expected to fall into each category, which is represented by each column in the confusion matrix. The numbers in each column represent the number of actual data points that match each class as predicted.

This paper is the first study to use multi-modal feature fusion method to detect OSAHS, so our confusion matrix is a 2-by-2 matrix. Each category corresponds to OSAHS and Normal.

## 4 Results and discussion

### 4.1 Results

Table 2 provides a comparative overview of the accuracy rates for different models used in experiments related to the detection of Obstructive Sleep Apnea-Hypopnea Syndrome (OSAHS) using electrocardiogram (ECG) signals as a basis for analysis. Among them, LSTM-SVM, RNN-SVM and CNN-SVM are classification models that integrate ECG signals features and biometric data features. LSTM, RNN and CNN only use ECG signals to classify OSAHS. The LSTM-SVM representative feature fusion model in this study has an accuracy of 97.1%, which is significantly better than other models.

**Table 2:** Experimental results for each model.

Model	Precision (Normal)	Precision (OSAHS)	Recall (Normal)	Recall (OSAHS)	F1-score (Normal)	F1-score (OSAHS)	Accuracy (%)
LSTM-SVM	100.0%	95.8%	91.7%	100.0%	95.7%	97.9%	97.1%
RNN-SVM	90.9%	91.7%	83.3%	95.7%	87.0%	93.6%	91.4%
CNN-SVM	76.9%	90.9%	83.3%	87.0%	80.0%	88.9%	85.7%
LSTM	64.3%	85.7%	75.0%	78.3%	69.2%	81.8%	77.1%
RNN	57.1%	81.0%	66.7%	73.9%	61.5%	77.3%	71.4%
CNN	50.0%	76.2%	58.3%	69.6%	53.8%	72.7%	65.7%

The results of the ablation experiment show that the traditional classification model is not effective in detecting OSAHS using ECG data alone. Among the models that classify ECG signals alone, the LSTM model with the highest accuracy only achieved an accuracy of 77.1%. However, in actual medical applications, this accuracy is still not enough to meet the need for reliable diagnosis. The results of the ablation experiment further highlight the key role of feature fusion in improving the overall performance of the model.

In the comparative experiment, we replaced LSTM with CNN and RNN respectively, and constructed CNN-SVM and RNN-SVM models to explore the performance of different depth models in feature extraction. The experimental results clearly show the superiority of LSTM in feature extraction. LSTM-SVM has outstanding performance in accuracy 97.1% and has achieved better results than RNN-SVM and CNN-SVM models. This shows that LSTM can capture information in input data more effectively and has a stronger ability to recognize complex patterns, thus providing more powerful feature support for the model. The introduction of LSTM not only improves the accuracy of the model, but also enhances its superiority in processing time-related features.

To further verify the model’s effectiveness on different patient groups and datasets, we applied the trained LSTM-SVM model to another independent dataset for verification. The results indicated that the model consistently maintained strong performance, achieving an accuracy of 92%, along with precision, recall, and F1 scores all at 95.7%. This demonstrates the model’s high generalization ability, as it not only excels on the training data but also effectively identifies and classifies samples in unfamiliar datasets. These findings highlight the robustness and adaptability of the model, showing its potential to perform reliably in varied clinical settings and patient populations. Nevertheless, we acknowledge that retrospective cross-dataset validation does not fully substitute for prospective real-world clinical evaluation. Future work will therefore focus on multi-center, prospective deployment studies and clinician-in-the-loop assessment to further establish workflow-level reliability and clinical utility.

Ultimately, the higher accuracy rates of the fusion models, specifically the RNN-SVM, and the exceptional performance of the ECG-TCN, underscore the efficacy of these advanced techniques in the challenging task of OSAHS identification and detection. These results suggest that a combination of temporal pattern recognition and salient feature extraction from ECG signals is a promising approach in the domain of medical detections for sleep disorders.

The F-scores of the model in this study are 95.7% and 97.9%, showing that our model performs well in terms of classification accuracy and breadth (as demonstrated in Table 1). It should be noted that the recall rate shows the model’s thoroughness, whereas the precision reflects the model’s accuracy. The F1-score that while aiming to enhance precision and recall, we also try to reduce their variation so that the F1-score can more properly judge the model’s strengths and flaws.

**Figure 3:**
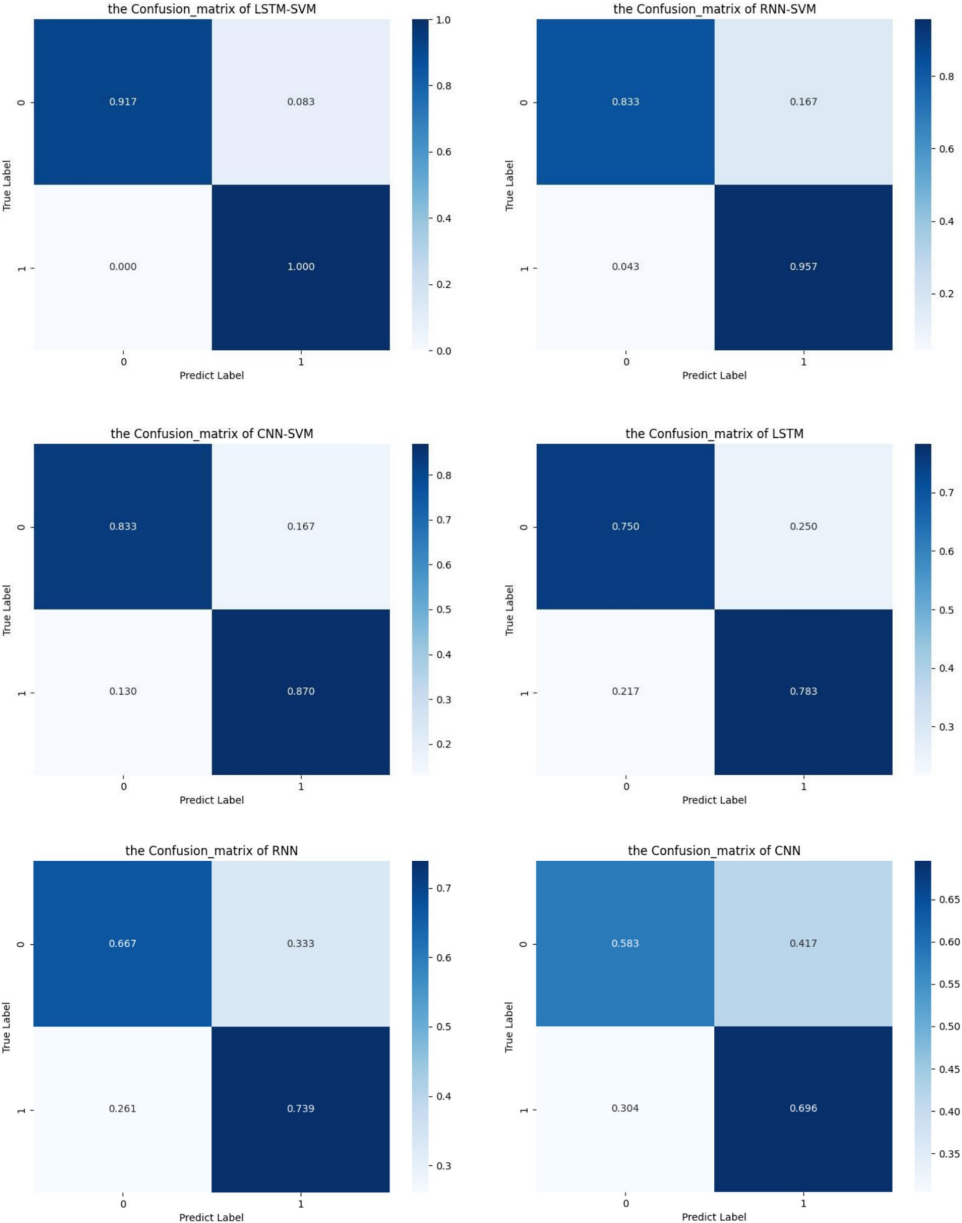
The confusion matrix of various models.

Figure 3 illustrates the confusion matrix for each model, demonstrating their respective classification performance. Among them, LSTM-SVM, RNN-SVM and CNN-SVM are classification models that integrate ECG signals features and biometric data features. LSTM, RNN and CNN only use ECG signals to classify OSAHS. Label 0 represents the Normal category, and label 1 represents the OSAHS category. Among them, our model’s prediction effect is very good in each category. In contrast, RNN-SVM performs poorly on the classification of Normal category, CNN-SVM performs averagely in classifying two categories. In addition, we can clearly see that it is very difficult to detect OSAHS by only classifying ECG signals. In stark contrast, our LSTM-SVM model stands out with an accuracy of up to 97.1%, outperforming all other models.

### 4.2 Discussion

In previous research, Cen et al. (48) employed deep learning techniques, specifically CNN, as a feature detector to learn the interrelationships between visible data and labels. The study connected the final fully connected layer of the CNN to the output layer for sleep apnea event classification. However, the experimental results indicated modest accuracy rates of 53.61 and 66.24% for hypopnea and apnea, respectively, suggesting limited practical applicability. Mashrur et al. (21) developed a new scale map-based CNN model to identify obstructive sleep apnea. A signal conversion pipeline was also created to translate ECG signals into OSA detection. Although its accuracy rate reaches 81.86%, it is still a long way from clinical application. There are also related studies on using biometric data to predict OSAHS risk through machine learning methods. Kuan et al. (25) established a model to detect OSAHS based on age, sex, and BMI, however, his study has several limitations. First, models trained solely on biometric data cannot be generalized well to other data sets, and his model does not include detailed anthropometric imaging or measurements, which may limit the model’s ability to distinguish disease-specific causes of OSAHS. In this study, we combined the characteristics of previous studies and fused the human body’s biometric data based on the anthropometric data ECG signals, and jointly used it for the detection of OSAHS. The combination of multimodal data in this study represents a significant innovation in OSAHS detection technology. It not only significantly improves detection accuracy, but also solves the limitations of previous single model approaches in specificity. This innovative approach also helps to unearth potential interactions between various biomarkers, providing a more comprehensive perspective on understanding the pathophysiology of OSAHS. In clinical applications, this fusion of multimodal data can improve the detection accuracy of OSAHS and help to optimize treatment plans. By combining ECG signals and biometric data, the patient’s pathophysiological state can be more accurately assessed, thereby providing support for clinical decision-making. Due to its high accuracy and specificity in diagnosing OSAHS, our model is well-suited for use in various medical institutions, including those with limited resources, such as primary care units lacking specialized physicians and advanced equipment. In the future, we should delve deeper into the potential clinical applications of this model. By integrating model outputs with Electronic Health Record (EHR) systems, we can assist physicians in clinical decision-making, thereby enhancing detection efficiency and accuracy. Furthermore, through automated data processing and analysis, the model can alleviate the burden of repetitive tasks for physicians, allowing them to focus more on complex clinical activities.

Although the proposed LSTM–SVM fusion network is a data-driven model, it provides meaningful avenues for clinical interpretation. The adaptive fusion weight w explicitly encodes the relative contribution of ECG-derived temporal representations and biometric risk attributes at the subject level. In our experiments, we observed that subjects presenting stronger apnea-related heart-rate variability patterns tended to receive higher ECG weights, whereas individuals with pronounced obesity-related anthropometric profiles (e.g., elevated BMI or body weight) exhibited greater reliance on biometric information. This behavior is consistent with the heterogeneous pathophysiological mechanisms of OSAHS and suggests that the model modulates its decision strategy according to clinically meaningful subject characteristics. Furthermore, inspection of the SVM coefficients indicated that BMI and body weight contributed most strongly to the decision margin, aligning with established epidemiological evidence linking obesity-related parameters to OSAHS severity. Taken together, these observations indicate that the model does not operate as a purely opaque black-box but instead integrates complementary physiological and anatomical information in a clinically plausible manner that may facilitate acceptance in real-world screening workflows.

With the popularity of mobile wearable devices and the continuous advancement of technology, individuals can now easily collect high-quality ECG signals by wearing smartwatches. Our model’s reliable performance ensures effective diagnoses even in such resource-constrained devices. The current work enables the integration of advanced health monitoring models into these devices, providing users with the convenience of monitoring their health status at any time. It not only helps users detect and treat potential health issues early, thereby reducing the burden on the healthcare system, but also allows for broad promotion due to the relatively low cost of the devices. In the future, our model can be used in mobile settings to achieve more efficient and accurate screening.

Although our model achieves higher accuracy than many previously reported approaches, cross-study performance comparisons should be interpreted with caution. Existing OSAHS detection studies differ substantially in terms of database selection, preprocessing strategies, label definitions, and evaluation protocols. In particular, a large proportion of prior work performs segment-level apnea event classification, whereas our study adopts a subject-level, record-based classification paradigm using full-night ECG recordings and biometric information. These methodological differences inevitably affect the numerical values of reported metrics and prevent strict one-to-one comparison across studies. Therefore, the comparisons presented in this work are intended to be contextual and qualitative rather than purely numerical, highlighting the relative advantages of multimodal fusion and subject-level modeling, rather than claiming direct superiority under an identical evaluation setting. Future work will incorporate unified benchmarking under harmonized preprocessing and validation protocols to enable more rigorous cross-method comparison.

One of the limitations of our model in future clinical applications stems from the availability of data. Although our model has been validated on two databases, it still lacks diversity, particularly in terms of data from Asian populations. This could potentially affect the predictive performance of the model. In the future, we aim to address this limitation by collaborating with hospitals and healthcare institutions to obtain more diverse datasets, thereby refining our model. Additionally, noise in ECG signals may adversely impact the accuracy of our predictions. To mitigate this, we plan to enhance our data preprocessing techniques in the future work, ensuring that useful information is preserved to the greatest extent possible.

## 5 Conclusion

In this paper, we propose a multi-modal feature fusion method (LSTM-SVM) for the detection of OSAHS. Furthermore, to effectively integrate ECG signals and biometric data, we propose a weighted feature fusion method with adaptive weights. To validate the effectiveness of LSTM-SVM, we conducted experiments on the Apnea-ECG Database. The results demonstrate that our LSTM-SVM model achieves an accuracy of 97.1% in classification. Comparing with previous classification models, our proposed model exhibits superior evaluation metrics, indicating significant improvements in the accuracy of OSAHS identification and detection. This advancement in diagnosis and detection of OSAHS offers enhanced reliability and convenience, enabling earlier stage diagnosis and treatment for patients while reducing the burden on healthcare professionals. This research can be expanded to cover more in-depth topics. For instance, by integrating mobile portable devices with sensor technology, our model empowers patients to detect OSAHS conveniently without the need for a hospital visit. Furthermore, the application of ECG signals detection technology can be extended to other disease diagnoses, facilitating the utilization of artificial intelligence technologies in the medical sector.

## Data Availability

The raw data supporting the conclusions of this article will be made available by the authors, without undue reservation.
