# Correction: Multimodal ECG and biometric data fusion for improved detection of obstructive sleep apnea hypopnea syndrome

**DOI:** 10.3389/fmed.2026.1794608

**Published:** 2026-01-28

**Authors:** Frontiers Production Office

**Affiliations:** Frontiers Media SA, Lausanne, Switzerland

**Keywords:** obstructive sleep apnea hypopnea syndrome, deep learning, electrocardiogram, LSTM, detection

Affiliations were incorrectly assigned to the authors. The author list previously appeared as:

Quanjing Zhu^1, 2, 3^, Mingqing Liang^4^, Xingxin Gong^1, 2, 3^, Yong He^1, 2, 3^ and Chao Mao^5^^*^

The correct affiliations in the author list appears below:

Quanjing Zhu^2, 3, 4^, Mingqing Liang^5^, Xingxin Gong^2, 3, 4^, Yong He^2, 3, 4^ and Chao Mao^1^^*^

The original version of this article has been updated.

